# Somali women's perceptions and experiences of pain and pain relief during childbirth in Norway: A qualitative study

**DOI:** 10.18332/ejm/176034

**Published:** 2024-02-05

**Authors:** Hanna Oommen, Leila Esse, Sanabel Sajer, Mirjam Lukasse

**Affiliations:** 1 Department of Obstetrics and Gynecology, Sørlandet Hospital Kristiansand, Kristiansand, Norway; 2 Center for Women's, Family and Child Health, Faculty of Health and Social Sciences, University of South-Eastern Norway, Kongsberg, Norway; 3 Department of Nursing and Health Promotion, Faculty of Health Sciences, Oslo Metropolitan University, Oslo, Norway; 4 Division of Obstetrics and Gynecology, Oslo University Hospital, Oslo, Norway

**Keywords:** pain, Norway, pain relief, Somali women, childbirth, epidural analgesia

## Abstract

**INTRODUCTION:**

Research shows that Somali women are less likely to receive epidural analgesia for labor pain compared to non-immigrant women in Norway. It is unclear why. This study aimed to explore the perceptions and experiences of Somali women regarding pain relief during childbirth.

**METHODS:**

In January 2023, semi-structured interviews were conducted with 10 Somali women who had lived in Norway for at least ten years and given birth within the past 12 months. Data were analyzed using thematic content analysis as described by Graneheim and Lundman.

**RESULTS:**

Three themes emerged from the data: a cultural understanding of epidural analgesia, religious and cultural coping strategies, and the midwife's role during labor. The participants expressed that there is a prevalent understanding in the Somali community that epidural analgesia in childbirth subsequently causes physical problems. Participants felt the influence of friends and family, expressed the need for culturally adapted information prior to labor, and concluded that, ultimately, what they decided upon was their own choice. They emphasized the belief that women were designed by God for childbirth. Prayer and placing oneself in God's hands were mentioned as pain management strategies. Nevertheless, the Somali women highlighted the importance of having a culturally competent midwife who is present when needed, creates trust, and provides information and access to different methods of pain relief, including epidural analgesia.

**CONCLUSIONS:**

Understanding Somali women's cultural and religious background while listening to individual needs for information and pain relief is essential to ensure a positive birth experience for women from a Somali background.

## INTRODUCTION

Migration and cultural diversity constitute a global challenge to public health services striving to provide high-quality, culturally appropriate care for all. In Norway, more than a quarter of the birthing population consists of immigrant women, including first- and second-generation immigrants^1^. Somali are one of the largest non-Western population groups in Norway, with most of the first-generation Somali immigrants being refugees^1,2^. Somali women have the highest fertility rate in the country, reflecting to some extent their cultural emphasis on the importance of a family structure with multiple children^1,3,4^. For many immigrant women, pregnancy and childbirth may be their first contact with the healthcare system and constitute their first hospital admission^5,6^.

Numerous Norwegian studies have shown that immigrant women, including Somali, have a higher risk of complications in pregnancy and childbirth, as well as poorer perinatal outcomes when compared to non-immigrant women^5,7^. These risks include pre-term birth, placental abruption, emergency cesarean section, stillbirth, and perinatal death^7-12^. Furthermore, Norwegian studies show that Somali women receive less epidural analgesia (EDA) during childbirth compared to both ethnic Norwegian and other immigrant women^10,13,14^. Additionally, Scandinavian studies show that immigrant women are less likely to use non-pharmacological pain relief in labor compared to non-immigrant women^15,16^.

Reduced use of pharmacological and non-pharmacological methods of pain relief may be due to a cultural perception of childbirth in which a natural approach is preferred, and there is a high level of confidence in the woman’s body to manage labor pain^16^. Alternatively, it may reflect immigrant women’s limited access to pain relief due to cultural and linguistic barriers, misinterpretation by staff of expressions of pain, women’s limited knowledge of pain relief options and misconceptions about side effects^13,16,17^.

The World Health Organization (WHO) recently incorporated the aspect of a positive experience in its childbirth care guidelines^18^. While experiencing pain during childbirth does not necessarily lead to a negative childbirth experience^19^, having adequate support and access to effective ways to cope with labor pain influences women’s experiences^6,20^. Studies from other areas of reproductive healthcare in Norway have shown that Somali women have difficulty accessing and understanding health information and available services, and often lack trust in the information provided^21,22^. Little is known about how Somali women in Norway experience intrapartum care and childbirth. Our study aimed to explore Somali women’s perceptions and experiences of pain and pain relief in labor.

## METHODS

In this qualitative study, we interviewed 10 Somali women. Participants were recruited through social media platforms such as Facebook and Snapchat, in groups and chats known to be frequented by Somali women^23^. The posts on these platforms provided an overview of the project’s purpose, methodology, consent process, anonymity measures, inclusion criteria, and contact information.

To be eligible for participation, women had to be of Somali descent, have delivered a child in a Norwegian hospital within the past 12 months (with at least six months having passed since childbirth), and have proficiency in the Norwegian language. The reason for this was to avoid language and communication issues being the major reason for the Somali women not receiving the pain relief desired, as these have been amply described previously. Additionally, they were required to have resided in Norway for a minimum of 10 years or been born in Norway with Somali parents. The rationale for this was to explore to what extent participants perceived the influence of Somali culture even after an extended time in Norway. Empirical and research evidence suggests that ethnic Somali strongly adhere to their native culture, and even among the second-generation acculturation may be limited^24^. Exclusion criteria included having given birth by elective cesarean section.

Fourteen women initially expressed interest, of whom four did not meet the inclusion criteria. Two participants withdrew before the interviews, and the last two participants were recruited through the snowball method, where one of our informants referred us to two other women^23^. Background information on the participants can be found in Table 1. All women were working, four of them in the health sector. One woman had an emergency cesarean section, while the others had vaginal deliveries.

**Table 1:** Background information on Somali women who had lived in Norway for at least ten years and given birth within the past 12 months (N=10)

*No.*	*Age (years)*	*Years lived in Norway*	*Number of children*	*Number born in Norway*	*Age of youngest (months)*	*Epidural analgesia last labor*	*Education*
1	37	37	3	3	11	No	Bachelor’s degree
2	28	28	1	1	6	Yes	Higher, but not Bachelor’s degree
3	30	16	2	2	6	Yes	Bachelor’s degree
4	33	22	2	2	12	Yes	Bachelor’s degree
5	33	14	4	4	7	No	Secondary degree
6	32	21	2	2	11	Yes	Bachelor’s degree
7	37	12	5	4	6	No	Bachelor’s degree
8	30	30	1	1	12	Yes	Bachelor’s degree
9	35	18	3	3	6	Yes	Secondary
10	34	16	2	2	7	No	Secondary

Data were collected through semi-structured interviews conducted in January 2023 by the second and third authors. The interviews lasted, on average, 29 minutes. Interview locations were chosen based on participants’ preferences: two at a coffee shop, two at their homes, and six via Zoom. All the interviews were audio recorded. To ensure that the participants covered the key topics essential for addressing our research questions, we prepared an interview guide.

### Analysis

The second and third authors conducted the interviews together. They wrote reflective notes immediately after the interviews and discussed the interviews with the last author. Subsequently, they transcribed the audio recordings verbatim into written texts, which were read and re-read several times by the team. The data were further analyzed using thematic data analysis, as described by Graneheim and Lundman^25^. This encompassed the iterative processes of identifying meaning units, condensing meaning units, assigning codes to these units, categorizing codes into categories and subcategories, assessing the findings, describing, and interpreting. We placed all meaning units in the first column of a table; in the second column, we condensed the text, the next column included codes, followed by a column for subcategories and categories (Supplementary file). The second and third authors assigned codes to condensed meaning units. The codes were categorized into groups that seemed to belong together. The researchers collaboratively reviewed and discussed the content of meaning units and codes against the emerging subcategories and categories in the table, paying particular attention to ensure that relevant information was not omitted. Finally, categories were described, and significant participant statements were extracted to illustrate the findings.

### Ethics

The study followed the Helsinki Protocol (WMA Declaration of Helsinki at www.wma.net). The study was approved by the Norwegian Agency for Shared Services in Education and Research (SIKT; Nr. 252727). The participants received both oral and written information and consent forms before the interview. Signed consent forms were obtained at the start of the interview. Any questions the women had about anonymity, privacy, and storage of the audio recording were answered.

## RESULTS

Thematic analysis revealed three main categories with ten subcategories (Table 2). The three main categories were: 1) A cultural perception of EDA, 2) Religious and cultural coping strategies, and 3) The midwife’s role during labor.

**Table 2:** The categories and subcategories

*Categories*	*Subcategories*
**A cultural perception of EDA**	EDA causes physical problems later
	The influence of the opinion of family and friends
	The need for factual and culturally adapted information about EDA before labor
	A personal decision despite cultural background
**Religious and cultural coping strategies**	Prayer, ‘dua’, and placing yourself in God’s hands
	God created women to tolerate birth
	Maintaining mental control and being quiet about the pain
**The midwife’s role during labor**	Physical and mental presence when required
	Relational and cultural competence
	Provide information and access to pain relief methods

### Cultural perception of epidural analgesia

*Epidural analgesia causes physical problems later*

Participants reported the widespread perception within the Somali community that having an EDA during labor would result in subsequent back pain. The apprehension about the effects and side effects was transmitted by their mothers who had given birth in Somalia, where this form of pain relief was not available. One woman also mentioned that having an EDA could cause menstrual pain, while another had heard of a risk of paralysis. As seen in the quote below, one participant attributed her own back pain to having had an EDA during labor and regretted her choice. Temporary relief was to be weighed up against long-term physical consequences:

*‘Mothers who gave birth before me, yes … and some say they got pain, or they still have back issues. They struggle during their menstrual cycles, and they blame it on getting the coil or whatever this thing is called … that’s placed on the back [EDA catheter]. I didn’t want that … and your back you need when the ordinary days come … and when you have heard that you will have back problems or problems with your period … well so … yes, no … I did not want to struggle afterwards.’* (Interview 5)

*The influence of family and friends*

The opinions of family and friends influenced the participants’ attitudes towards EDA. All of the women had been advised against having an EDA. They were told that pain relief is unnecessary because pain should be endured and that Somali women should be able to manage without EDA. Most participants conveyed that their mother had the strongest influence on their thoughts about EDA. For example, one woman’s mother – who was with her during labor – told her to ‘put on a brave face and think of the women in Somalia’. The participants’ mothers had all given birth without EDA, and the participants considered them to be role models. The presence of their mothers during childbirth, offering encouragement to endure the process, as Somali women traditionally do back in their home country, helped several women to endure:

*‘I’m the type who believes in gritting your teeth and enduring. I think of mothers … my mother, my grandmother who gave birth in Somalia. Without anything. I thought, “If my mom can do it, I can, too”.’* (Interview 1)

Some of the women expressed experiencing a sense of pride and achievement in giving birth without the use of EDA.

*The need for factual and culturally adapted information about EDA before labor*

Participants pointed out that they had limited knowledge about available pain relief methods. They perceived that a language barrier was an important reason for this. In addition, they spoke of Somali women sharing incorrect information about EDA with each other, adding to the already present skepticism against pain relief. They agreed that the correct way to eradicate misunderstandings and false information was through appropriate, understandable, factual information from healthcare staff. Several of the participants had actively sought information regarding the side effects of EDA, often consulting with healthcare professionals. A few of the participants were healthcare staff themselves and reported that their increased knowledge was a benefit:

*‘I know many who can’t handle childbirth pain like I can … and I think a lot about how they managed, how? … I had the opportunity to learn Norwegian and study and get the right information compared to others, and there’s a lack of knowledge.’* (Interview 7)

The participants expressed a need for better, culturally tailored information on pain relief options during pregnancy, preferably by Somali midwives in a group session with other Somali women, to stop what they called the ‘vicious circle of misinformation’.

*A personal decision despite cultural background*

Despite the rumors of the potential adverse effects of having an EDA during labor and the influence of friends and family, several women ultimately opted to have it as a form of pain relief. These women described their labor pains as extreme and uncontrollable, and most had made the decision to have an EDA prior to going into labor. They knew it was the best thing for them and did not allow their mother or cultural opinions about EDA to dominate their thoughts or actions:

*‘Should I listen to my mom saying, “Yes, don’t take it?” No. Why should I use the ball? I’ve expended so much energy, and no.’* (Interview 3)

These women experienced minimal objections from their mothers. It was clear that it was their decision. In their view, women in Norway, themselves included, are privileged to have access to adequate pain relief, and they value their own judgment over cultural beliefs.

### Religious and cultural coping strategies

*Prayer (dua), placing yourself in God’s hands*

All participating women spontaneously mentioned that they needed Allah’s help to manage labor. They actively sought His help through prayer or ‘dua’ (supplication). They witnessed that this coping strategy brought them inner peace and strength as well as significantly eased their pain and provided comfort. Women believed that their prayers were answered and provided the needed help:

*‘It somewhat eased my pain; every time I had pain, I prayed to God, and my whole mindset was connected to God, relieved … “dua” [personal supplication to God] that helps when you have pain and so on … I’ve read a lot about I t… That was very, it helps, you know. Now, I have God with me, no matter what.’* (Interview 10)

These rituals were seen as a sign of reliance on Allah and contributed to a sense of security. Most women in the study believed that Allah’s presence significantly influenced how they coped with pain during childbirth. They felt that they had no control over the birthing process and that it was solely in Allah’s hands. Actively surrendering to Allah’s will was seen as a form of pain relief. As one woman expressed:

*‘When I place all my faith in God… that I trust in you, Allah, and I need your help … and I find that very comforting. To have that faith and surrender to God’s will … that I am your servant, and I need you. So, I received the help I needed from God, in my opinion.’* (Interview 5)

*God created women to tolerate birth*

Participants expressed a strong belief that Allah created women to give birth. This trust that Allah had designed women to be able to bear childbirth was important. Their belief in this divine purpose, rooted in cultural and religious traditions, provided them with the strength and determination to endure the challenges of childbirth:

*‘So, going through childbirth, you earn a lot of “ajjer” [reward from God]. And so much good and merit from it, so for me, when I felt pain, I just held it inside and thought, “Ahh … I can handle this … Allah never gives you something you can’t handle.’* (Interview 8)

Another participant believed that if she died during childbirth, she would go to heaven, which helped her endure the pain, as she saw giving birth as a significant reward from God. A few women mentioned that their labor pains and childbirth could be perceived as a trial from Allah.

*Maintaining mental control and being quiet about the pain*

The women stressed the importance of mental control and avoiding anything that might impair it, such as nitrous oxide gas. They believed it could affect their awareness during childbirth. Some women found that feeling the pain was a positive aspect of the birthing experience, as it allowed them to stay alert and engaged in the process, even declining an epidural for this reason:

*‘Actually … even though contractions are very painful … childbirth is very painful, I think it’s a very nice experience, feeling everything that’s happening, following the progression of childbirth, and giving birth.’* (Interview 5)

Participants explained that, according to Somali culture, women should be silent when experiencing pain and that this is a tradition instilled from childhood. Expressing pain loudly is discouraged, and complaining about pain is considered shameful. Mothers also influence how their daughters express pain during childbirth, teaching them to endure silently. This was not just a negative thing, not expressing pain, but also part of who women perceived they were as a person and where they belonged, part of an ongoing line of Somali women:

*‘I find giving birth very demanding. It’s very painful for me; I get really bad back pain. But it has also become a part of the culture because I grew up learning not to express pain. It really affects who we are, personally. It’s these kinds of things that shape who we actually are.’* (Interview 7)

### The midwife’s role during labor

*Physical and mental presence when required*

In the interviews, the women highlighted the importance of having the midwife physically present. They experienced less fear when the midwife was in the room. When the midwife left the room, they did not cope as well with the contractions. In addition, some women spoke about how, even if the midwife was physically present (checking up on things), sometimes they seemed unapproachable. While participants understood that midwives had other demands on their time, several wondered why their midwife appeared to be with them less than with others. They wondered if it was related to their Somali background or how they expressed or rather did not express their pain. As one woman said:

*‘She prioritized her because she was younger and because she was White.’* (Interview 2)

Women did not use the term discrimination. However, it was clear they were aware of their own ethnic background being very visible and different from most other women, and considered that this could influence how much time the midwife spent with them.

*Relational and cultural competence*

All of the participants commented on how they experienced their midwifery care. They wanted a reassuring midwife who instilled trust and treated them as a person, not just a childbearing woman. Participants emphasized the importance of good communication and appreciated physical touch. Some reported that shift changes and the need to build new relationships with different midwives were challenging and frustrating:

*‘The reason I didn’t trust the midwife was because I had three different ones … So with the first one, I opened up a bit, and then she disappeared, and another one came, and then she disappeared too, and I thought, “Ahh … I don’t need to open up to all of them, just let them do their job, and I actually have my husband here. If my husband wasn’t there, it would be a different story. Then, I would have opened up to them.’* (Interview 8)

Midwives’ lack of cultural understanding regarding how (or whether) Somali women express pain resulted in inadequate pain relief and frustration among some women. Women tried to be as quiet as possible and not express or verbalize their pain at all. This could be interpreted as them coping very well with the pain. As one woman said:

*‘I hoped she would understand that I was in pain. I just moaned and turned back and forth.’* (Interview 10)

Women expressed the desire for a culturally knowledgeable midwife and more culturally diverse staff. A couple of women met a midwife whose outward appearance showed that she was not ethnic Norwegian and really appreciated that even if the midwife was not Somali.

*Provide information and access to pain relief methods*

Women expressed their concern that ethnic Norwegian midwives would meet them with a stereotyped understanding that Somali women do not want any form of pain relief. Women sought midwives who were culturally aware and capable of addressing misconceptions surrounding EDA, thus refuting any concerns held by them and their families. They did not want midwives’ (lack of) cultural understanding to result in the midwives not offering EDA and/or other methods of pain relief. They wanted timely information and the ability to make informed decisions about their own pain relief. Women appreciated being offered alternative methods to EDA, such as massage, changing positions, and water immersion:

*‘I could not move … she helped me to move a little. She gave me different positions, and that helped against the pain, so I thought that was very good.’* (Interview 2)

Breathing and relaxation techniques assist women in maintaining mental control. However, in some interviews, participants mentioned having had limited awareness of non-pharmacological pain relief methods and the need for more information and access to these methods.

## DISCUSSION

This study explored Somali women’s perceptions and experiences of pain and pain relief in labor. Three main themes were identified: the cultural perception of EDA, religious and cultural coping strategies, and the role of the midwife.

The women in our study conveyed skepticism against EDA. Women expressed fear of long-term physical consequences, such as lasting backache. This apprehension was transmitted by their mothers, who had given birth in Somalia, where this form of pain relief was not an option. Furthermore, Somali women in the current generation associate some of their physical discomfort with having had an EDA, reinforcing the prevailing cultural perspective. The Somali women in another study were also found to believe that EDA would prolong labor^26^. Large quantitative studies show that Somali women receive less EDA as well as other methods of pain relief^13,14^. This underuse may reflect Somali women’s own choices, as the findings in our study suggest. However, limited use of EDA stems from insufficient awareness about its effects, and evidence-based information on side-effects initiatives is necessary to enhance informed decision-making.

Our study highlights the pivotal role of cultural perceptions in shaping women’s choice of pain relief methods during childbirth. Culture is defined as ‘the collective programming of the mind that distinguishes the members of one group or category of people from another’ and can be categorized into individualistic versus collective^27^. Individualistic cultures, as Norway has been described as having, prioritize personal interests^26^. In a collective culture, like in Somalia, the person belongs to an in-group, such as their extended family, from which they cannot be detached. Individualism and collectivism have been found to influence decisionmaking^27^. Our findings demonstrate that the Somali women in our study belonged to a collective culture, as they were influenced by their mothers. However, a notable shift from a culturally collectivist perspective towards a more individualistic culture was apparent among participants, with women asserting their right to decide whether they wanted EDA.

The Somali women in our study acknowledged the severity of pain in childbirth, yet they perceived it as a positive form of suffering, serving the profound purpose of bringing new life into the world, as designed by Allah. This kind of meaning-making through spirituality and confidence in Allah has been described as aiding women with persevering through childbirth^28^. In line with previous research, our study found that the practice of remembrance of Allah, described as ‘dua’ (praying and supplication in our study), assisted women in maintaining self-control and alleviating anxiety in labor^17,28,29^.

As supported by previous research, the women in our study expressed the cultural belief within Somali culture that it is unacceptable to vocalize pain^17,29,30^; one result may be that Somali women may not receive the pain relief they require and desire. Establishing a relationship and being present seemed to be crucial for midwives’ ability to provide high-quality, individualized care, a finding consistent with previous research^30^. As we (and others) have found, another way to ensure that women receive their desired pain relief is to provide sufficient information about the available options prior to birth^17,30,31^. The women in our study who had some form of healthcare education were certain that this was key in their decision-making regarding which method of pain relief to use. However, a study among 302 Somali women in Oslo showed that 71% lacked the ability to obtain, understand, and act upon health information and services, and to make appropriate health decisions^21^.

The women in our study expressed the need for culturally sensitive and competent midwives. These skills have been highlighted in the evidence-based, informed framework for maternal and newborn care presented in Renfrew et al.^32^. As our findings indicate, it may be easier to achieve culturally sensitive and competent care with healthcare staff who have a similar cultural background. In other studies, as in ours, Somali women perceived they were treated differently because of their ethnic background and/or inability to communicate well with staff^31^. This finding is important, as women’s perception of being discriminated against can have adverse effects on the childbirth experience and on birth-related outcomes^33^.

### Strengths and limitations

Somali women have been described as ‘hard to reach’ and recruited into research studies, but one of the authors is of Somali background, and this helped the recruitment process^23,34^. Another strength of our study was the visible cultural nearness of the interviewers to the participants; both interviewers were immigrants with a Muslim background, one of whom was of Somali descent. This may have made the participants feel more at ease and willing to share their experiences; one interviewer even occasionally spoke Somali, increasing the trustworthiness of the findings. Another strength is that neither of the interviewers had a professional relationship as carers of the participants.

A limitation of the study concerns the fact that the Somali women included in our study had been living in Norway for at least ten years. The transferability of our findings is thus limited with regard to recently immigrated women. Another potential limitation is that to reach sufficient participation, we included two women who had given birth twelve months prior to the interview. Recall bias may, therefore, have influenced the findings. However, research suggests that women have little difficulty remembering their childbirth experience^35^. Finally, we did not ask the women specifically who accompanied them in childbirth and how this influenced their experience of pain and pain relief. In Somali culture, men do not traditionally accompany their wives in labor, and their presence may have affected the pain relief the women requested or their experience of support^29^.

## CONCLUSIONS

This study demonstrated the influence of Somali women’s cultural background on their experience of pain and pain relief in labor. The women had a culturally informed view of EDA, used cultural and religious coping strategies, and desired culturally sensitive care from their midwives. This study reminds us that childbirth is not only a biological process but also a social and cultural process requiring caregivers who can combine clinical knowledge and skills with interpersonal and cultural competence.

## Data Availability

The data supporting this research are available from the authors on reasonable request.
