# Supplementary material for: Somali women's perceptions and experiences of pain and pain relief during childbirth in Norway: A qualitative study
Source: Eur J Midwifery. 2024 Feb 5;8:10.18332/ejm/176034. doi: 10.18332/ejm/176034 (PMC10845056; doi:10.18332/ejm/176034)
Supplement: Supplementary file 1 [file EJM-8-05-s1.pdf]

Supplementary file

Example of analysis

Meaning unit	Condensed meaning unit	code	Subcategory	Category
“There are many Somalian women who think that because of the epidural you can have later side-effects like backache and really seriously they believe this”	Somalian women think EDA has side-effect like backache	Backache because EDA	EDA causes physical problems later	A cultural perception of EDA
“… and I am like strong in believing in that this is part of what Allah has given a woman, this with giving birth!	Strong belief that Allah has given women the task to give birth	Created for childbirth	God has created women to tolerate birth	Religious and cultural coping strategies

© 2024 Oommen H. et al.
